# Emerging Role of C5 Complement Pathway in Peripheral Neuropathies: Current Treatments and Future Perspectives

**DOI:** 10.3390/biomedicines9040399

**Published:** 2021-04-07

**Authors:** Cristina Giorgio, Mara Zippoli, Pasquale Cocchiaro, Vanessa Castelli, Giustino Varrassi, Andrea Aramini, Marcello Allegretti, Laura Brandolini, Maria Candida Cesta

**Affiliations:** 1 Dompé Farmaceutici SpA, 80131 Napoli, Italy; cristina.giorgio@dompe.com (C.G.); mara.zippoli@dompe.com (M.Z.); pasquale.cocchiaro@dompe.com (P.C.); 2 Department of Life, Health and Environmental Sciences, University of L’Aquila, 67100 Coppito, Italy; vanessa.castelli@univaq.it; 3 Paolo Procacci Foundation, 00193 Rome, Italy; giuvarr@gmail.com; 4 Dompé Farmaceutici SpA, 67100 L’Aquila, Italy; andrea.aramini@dompe.com (A.A.); marcello.allegretti@dompe.com (M.A.); laura.brandolini@dompe.com (L.B.)

**Keywords:** complement system, C5a/C5aR axis, C5a receptor1, peripheral neuropathy, pain, C5aR inhibitor, allosteric modulator

## Abstract

The complement system is a key component of innate immunity since it plays a critical role in inflammation and defense against common pathogens. However, an inappropriate activation of the complement system is involved in numerous disorders, including peripheral neuropathies. Current strategies for neuropathy-related pain fail to achieve adequate pain relief, and although several therapies are used to alleviate symptoms, approved disease-modifying treatments are unavailable. This urgent medical need is driving the development of therapeutic agents for this condition, and special emphasis is given to complement-targeting approaches. Recent evidence has underscored the importance of complement component C5a and its receptor C5aR1 in inflammatory and neuropathic pain, indicating that C5a/C5aR1 axis activation triggers a cascade of events involved in pathophysiology of peripheral neuropathy and painful neuro-inflammatory states. However, the underlying pathophysiological mechanisms of this signaling in peripheral neuropathy are not fully known. Here, we provide an overview of complement pathways and major components associated with dysregulated complement activation in peripheral neuropathy, and of drugs under development targeting the C5 system. C5/C5aR1 axis modulators could represent a new strategy to treat complement-related peripheral neuropathies. Specifically, we describe novel C5aR allosteric modulators, which may potentially become new tools in the therapeutic armory against neuropathic pain.

## 1. Introduction

The complement system is a crucial element of the innate immune response that works in concert with antibodies and phagocytic cells to clear pathogens [1]. It consists of a number of precursor proteins that are cleaved by specific proteases to generate various complement peptides and fragments, ultimately leading to the formation of the Membrane Attack Complex (MAC) [2]. One of the key components of the complement system is the Complement 5 (C5) protein, the cleavage of which is mediated by the serine proteases C5 convertases to generate two different fragments [3]: C5a, which is a potent chemoattractant and pro-inflammatory modulator [4,5], and C5b, which initiates the formation of MAC, mediating cell lysis and triggering inflammation [6]. When properly activated, C5a is crucial for host defence system and clearance of pathogens; however, its inappropriate activation is involved in a wide range of disorders, including peripheral neuropathic diseases [7,8].

Here, we provide an overview of complement pathways and key components that are associated with the dysregulated complement activation in the onset and progression of peripheral neuropathies. Specifically, we describe the involvement of C5a and C5a receptor 1 (C5aR1) signalling in several peripheral neuropathies, such as Guillain-Barré syndrome (GBS), chronic inflammatory demyelinating polyradiculoneuropathy (CIDP), familial amyloid polyneuropathy (FAP), and chemotherapy-induced peripheral neuropathy (CIPN). We also discuss emerging anti C5-targeted therapies, including monoclonal antibodies, peptidomimetics and small molecules that are currently under pre-clinical development or that are already used in clinical practice, highlighting how anti-C5 treatments may provide an alternative and selective approach to the challenging treatment of neurological conditions that respond poorly to current therapies.

## 2. Complement Pathways

The complement is a major component of the innate immune system and acts as a bridge between innate and acquired immunity. Over the years, it has become clear that the complement has various functions, ranging from the mediation of inflammatory responses to the regulation of host cell clearance after their programmed cell death [9], and takes part in nearly every step of the immune reaction. It is composed of over 50 proteins [10]. Among these, the soluble ones are produced mainly by the liver and can be detected in the plasma and on cell surfaces as inactive precursors (zymogens) [1]; their cleavage by serine proteases activates a cascade of enzymatic reactions that is tightly regulated to assure complement activation is triggered only at specific locations, thus avoiding host tissue damage.

**Figure 1:**
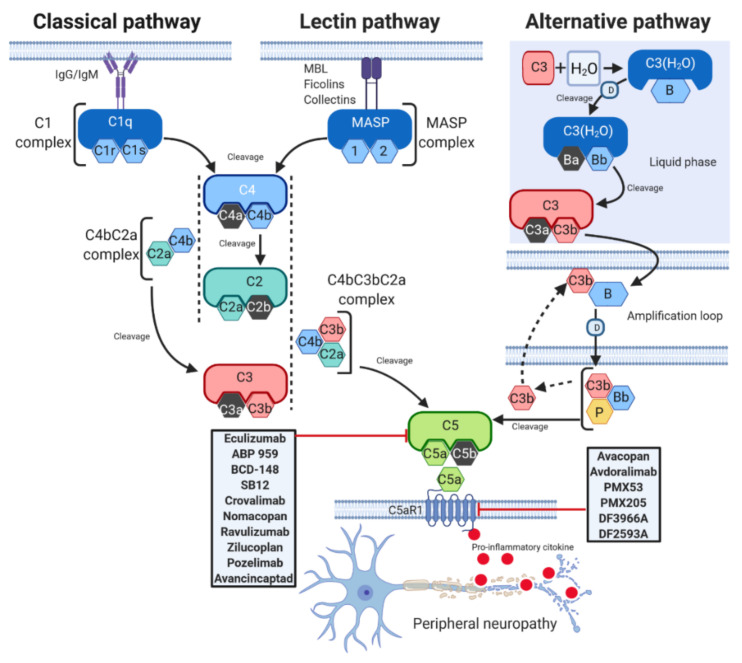
C5a complement activation pathways. The C5a complement system can be activated through three pathways: classical (CP), lectin (LP), and alternative (AP). CP begins with antibody-mediated activation of C1 complex, which leads to formation of the C4bC2a complex, the C3 convertase. This C3 convertase cleaves C3 to produce C3b, which forms a complex with C4b and C2a. This complex is the C5 convertase, which cleaves C5 to produce C5a and C5b. The LP begins with signal recognition by oligomeric structures of mannose- binding lectin (MBL), ficolins and collectins, which activate mannan-binding lectin serine proteases (MASP) 1 and 2, which in turn mediate the production of C4b. From this point, the LP follows the same steps as the CP. In the AP, C3 interacts with factor B (B) and factor D (D), leading to cleavage of further C3, and this process is perpetuated through an amplification loop. In the final step of this pathway, even properdin (P) is involved. Additional C3b binds to the C3 convertase and forms a C5 convertase, which cleaves C5 to form C5a and C5b. C5a activates on C5aR1, a prototypical G-protein coupled receptor (GPCR) recruiting immune cells to the site of inflammation. Drugs targeting C5 or C5aR1 in different stage of development are reported in the gray rectangles. T-arrow indicates inhibition of pathway at point of intersection.

Activation of the complement system occurs through three distinct pathways: the classical (CP), lectin (LP), and alternative (AP) pathways [2] (Figure 1).

Although each of them is differentially initiated and is characterised by unique and specific factors, they all include the activation of C3 and C5 and lead to a common pathway, which results in the formation of the MAC, ultimately inducing cell lysis by binding to the target cell membrane [11]. The CP is activated by the binding between Immunoglobulins M or G (IgM or IgG), and several other proteins such as C-reactive protein and serum amyloid P protein [12], and C1 complex, which is constituted by the sensing molecule C1q and two heterodimers formed by the zymogens C1r and C1s [12]. C1q activates C1r, which in turn cleaves C1s [13]. Once activated, C1 enzyme complex mediates the cleavage of native C4, which is followed by cleavage of C2 and the subsequent formation of the C3 convertase C4bC2a. The C3 convertase activates C3, triggering the dissociation of C3 into C3a and C3b. C3b then binds to the existing C3 convertase to form the C5 convertase C4bC3bC2a complex, which cleaves C5 to generate two different fragments, namely C5a and C5b [14]. The LP is very similar to the CP. The activated LP complex has an oligomer structure similar to the pentamolecular C1 complex [15] and is triggered by serine proteases associated with mannose-binding lectins (MBLs) and with ficolins, another family of lectins, which are able to recognize pathogens [16]. Upon activation by these signals, the enzymes of the complex mannan-binding lectin serine protease (MASP) 1 and 2 mediate the formation of the C3 convertase C4bC2a, which activates the same downstream pathways as occurs in CP [17]. In contrast to the classic and lectin pathways, the AP can autoactivate using a process termed “tickover” of C3 [18]. Initially, a spontaneously generated thioester- hydrolysed form of C3 (C3(H_2_O)) interacts with factor B and factor D to form the fluid phase alternative pathway C3 convertase C3(H_2_O)Bb [19]. C3(H_2_O)Bb cleaves native C3 to generate C3a and C3b, and the latter binds to its receptor on the lipid membrane. On the membrane, C3b combines with factor B, which is cleaved by factor D to form the alternative pathway C3 convertase C3bBb [14]. Properdin (P), a positive regulator of the complement system, stabilizes C3bBb, and the binding of additional C3b (amplification loop) to the existing alternative pathway C3 convertase generates a C5 convertase, thus leading to the production of C5a and C5b [14].

As it is a very complex system, various mechanisms can interfere with the complement cascade leading to over-activation and consequent neuronal damage and disease [20]. Dysregulated complement mechanisms, such as those involving C5 components, contribute to the initiation and progression of several neuropathies [21,22].

## 3. Peripheral Neuropathies and C5a/C5aR1 Axis

The term “neuropathy”, or also peripheral neuropathy (PN), refers to a group of conditions characterized by damage and loss of function of nerve cells in the brain or peripheral nervous system (PNS). The population prevalence is about 2400 per 100,000 rising with age to 8000 per 100,000 [23]. Although the damage occurs most frequently in the PNS, also brain injuries, such as stroke, can result in neuropathic symptoms [24]. Moreover, neuropathies can be expressions of neurodegenerative diseases [25], where the degeneration of sensory nerve fibres is due to a wide variety of insults, including diabetes, infectious diseases and nutritional deficiencies, and chemotherapy treatments [26]. Symptoms usually include numbness and paresthesia and are often accompanied by weakness and pain [27,28].

So far, progress in developing treatments for neuropathies has been frustratingly slow. In fact, despite the availability of therapies that can alleviate symptoms—as, for example, in the case of mild pain, which may be relieved by over the counter analgesics and topical patches—and can address conditions associated with PN [29,30,31,32], no treatments have been approved to date that directly modulate the underlying mechanisms of neuropathies. Current pharmacological therapies are only partially effective, and prolonged exposure to such agents can cause unwanted side effects. Consequently, there is an urgent need to identify and label specific molecular targets and to develop agents to treat pain by exploiting alternative biological pathways.

Over the last few years, evidence has indicated that C5a activation triggers a cascade of events that are involved in the pathophysiology of PN and in the genesis of painful states of neuro-inflammation [8]. C5a exerts its biological functions by binding two receptors, C5a receptor-like 1 (C5aR1, also referred to as CD88), a class A seven-transmembrane G-protein-coupled receptor (GPCR), and C5a receptor-like 2 (C5aR2, also known as C5L2 or GPR77) [33], a homolog of C5aR1, but which is not coupled to intracellular heterotrimeric G-proteins due to a mutation in G-protein recognition sequence. C5aR1 is expressed by a broad range of cell types, including all cells of myeloid origin (neutrophils, eosinophils, monocytes, macrophages, dendritic cells, mast cells), lymphocytes, and non-myeloid cells, such as lung, liver, kidney, skin, and central nervous system (CNS) cells [5,34]. C5aR2 is highly expressed in human tissues, such as bone marrow, spleen, and lung, as well as in immune cells, including most myeloid cells and specific T cell subsets [35]. C5aR1 is well-known for its pro-inflammatory effect [36]; conversely, the role of C5aR2 is poorly understood and still controversial [37]. Although C5aR2 can independently induce and modulate C5a biological functions through α-arrestin signalling, further investigations are needed to better understand its actual role [38].

By contrast, the C5a/C5aR1 axis triggers leukocyte recruitment and pro-inflammatory cytokines production, which drive inflammatory and neuropathic pain [39,40,41]. Up-regulated levels of C5a and C5aR were found in spinal cord microglia in animals subjected to spared nerve injury (SNI), a model of neuropathic pain [42], while local activation of C5aR1 was found to be implicated in the mechanical nociceptive sensitization in an in vivo model of postoperative pain [43]. In a similar model, PMX-53, a C5aR1 antagonist, decreased mechanical allodynia, oedema, and the levels of several inflammatory mediators present in incised skin [44]. Moreover, local pre-treatment of rats with PMX-53, attenuated mechanical hyperalgesia induced by zymosan, carrageenan, lipopolysaccharide, and ovalbumin, suggesting its role in the control of inflammatory pain [45]. In addition, oral administration of DF2593A, a non-competitive allosteric C5a inhibitor, effectively reduced mechanical hyperalgesia in a carrageenan and complete Freund’s adjuvant-induced inflammatory pain model. Furthermore, DF2593A reduced mechanical hypersensitivity in a model of neuropathic pain induced by SNI [40]. Notably, C5aR1 disruption in knock-out (KO) mice suppressed thermal hyperalgesia compared to wild-type (WT) mice and decreased mechanical sensitization after paw incision [40,41,43], suggesting a major involvement of C5a/C5aR1 axis in pain and inflammation after surgery.

In sum, the administration of C5aR1 antagonists produce analgesic effects in various models of inflammatory and neuropathic pain, highlighting the therapeutic potential of pharmacologically targeting the C5a/C5aR1 axis for chronic pain management.

## 4. Disease of the Peripheral Nervous System (PNS)

The role of C5a/C5aR axis activation in pain generation in neuropathies has been widely investigated in several pharmacological studies. The following paragraphs describe the more recent major findings in the field of PNS and discuss possible implications of the complement system in the pathogenesis of different neuropathic disorders.

### 4.1. Guillain-Barré Syndrome

GBS is a clinically heterogeneous spectrum of rare post-infectious neuropathies that usually occur in otherwise healthy patients and encompasses acute inflammatory demyelinating polyradiculoneuropathy (AIDP), acute motor axonal neuropathy (AMAN), acute motor-sensory axonal neuropathy (AMSAN), Miller–Fisher syndrome (MFS) and some other regional variants [46,47,48]. GBS is estimated to affect about 1 in 100,000 people each year and it can strike at any age and both sexes [49]. The exact cause of GBS is not known; it is characterized by symptoms that often affect the arms, breathing muscles, and even the face, reflecting widespread nerve damage. Several pathologic and etiologic subtypes of GBS exist, and in many cases it develops subsequently to minor infections but is not associated with other autoimmune or systemic disorders. Usually, GBS occurs after an infectious disease, during which antibodies that cross-react with gangliosides at nerve membranes-with anti-GQ1b ganglioside antibodies being the principal biomarkers of GBS [50]—are aberrantly generated and directed against the PNS, causing nerve damage or impairment of nerve conduction [51]. Anti-GQ1b ganglioside antibodies are principal biomarkers of GBS [50]. The concept of infection-triggered antibody cross-reactivity is well established in axonal GBS and this mechanism is suspected to play a key role in demyelinating GBS. Intravenous administration of immunoglobulins and plasma exchange are effective in treating GBS [52,53]; other therapeutic strategies have been tested in animal models, but their bench-to-bedside transfer is still lacking [54].

Inhibition of C5 complement component activation in experimental ex vivo and in vivo GBS models was extensively used to investigate the pathogenesis of GBS and to evaluate complement deposition in the nerve membrane [55,56,57,58,59]. Specifically, the complement inhibitor APT070 (Mirococept), which regulates C5 and C3 convertases, was shown to be efficacious in an anti-GQ1b-mediated mouse model of the GBS variant MFS, inhibiting the formation of MAC complexes and protecting nerve terminals [57]. Similarly, the anti-C5a monoclonal antibody eculizumab, which inhibits formation of C5a and C5b-9, was reported to prevent complement damage and respiratory paralysis in another severe in vivo mouse model of MFS generated via anti-GQ1b antibody and normal human serum injection as a complement source [58]. Together, these findings have raised the possibility of developing clinical trials using anti-C5a in GBS and in other antibody-mediated terminal motor neuropathies involving complement activation.

### 4.2. Chronic Inflammatory Demyelinating Polyradiculoneuropathy

CIDP is the most common chronic inflammatory neuropathy, and it is usually characterized by slow progressive, symmetric, proximal and distal paresis and sensory dysfunction. Symptoms develop in few months and the disease course can be either chronically progressive or relapsing with stepwise progression [60]. Prevalence is about 1 in 200,000 in children and 1–7 in 100,000 in adults, but it is recently accepted that the frequency is underestimated [61]. Although CIDP has been classified as an autoimmune disorder, in which an aberrant immune response is directed towards components of peripheral nerves causing segmental and multifocal demyelination, axonal degeneration and perivascular or endoneurial inflammatory infiltrates of macrophages and T cells, the exact mechanisms underlying the development of its immunopathology is still far from to be defined.

Individuals with CIDP lack a detectable antibody titer specific for major compact myelin proteins, thus suggesting that serum constituents, such as cytokines or components of the complement cascade, rather than myelin-directed antibodies might contribute to peripheral nerve injury [60,62,63]. Supporting the hypothesis that complement activation can be a potential pathogenic mechanism for this disease, complement component C3d deposition has been detected on the outer surface of PNS Schwann cells in biopsies from patients with CIDP [60,64,65]. Moreover, clinical studies demonstrated that CIDP patients have increased serum and cerebrospinal fluid levels of C5a [66], which is the result of the proinflammatory function of C3d aimed at recruiting myeloid cells, such as macrophages, to inflammation sites through complement receptors and inducing tissue injury through formation of the MAC. These findings suggest that systemic and local terminal complement activation is a characteristic feature of inflammatory demyelinating polyneuropathies and support a role of complement activation in the pathogenesis of CIDP.

### 4.3. Familial Amyloid Polyneuropathy

FAP, or transthyretin (TTR) amyloid polyneuropathy, is a progressive sensorimotor and autonomic neuropathy of adult onset, which is characterized by systemic accumulation of amyloid fibrils constituted of aberrant TTR protein [67]. The global prevalence is unknown, but in Japan it has been recently estimated to be around 1 person per million in the general population [68]. FAP is a heterogeneous disorder with a clinical presentation that varies based on the genotype and geographic origin [69,70]. To date, more than 40 TTR mutations have been identified and associated with different patterns of organ involvement, age of onset and disease progression [71,72]. The most common type of mutation is a substitution of valine for methionine at position 30 (ATTRV30M) [73]. The symptoms depend on the site of protein accumulation in the body and, although each TTR variant leads to a different phenotype, PN and cardiomyopathy are predominant hallmarks [74]. The disease usually worsens over 5 to 15 years, and often leads to death caused by heart failure due to TTR protein deposits. Liver transplantation is currently the only treatment for preventing synthesis of the amyloidogenic variants of TTR [75].

Nerve biopsies of individuals with amyloidogenic TTR revealed that in amyloid deposits, transthyretin is aggregated with several other proteins, such as apolipoprotein E, serum amyloid P, and complement C1q [76], suggesting a role for C1q in the pathogenesis of the disease. C1q protein has been shown to be involved also in other amyloidosis, such as Alzheimer’s disease, activating the complement pathway leading to neuronal loss [77]. It is speculated that the complement plays a dual role: although it is known that C1q is able to exert a neuroprotective function against toxic concentrations of soluble pre-amyloid aggregates [78,79], C5a is recognized as having a detrimental neuro-inflammatory effect [80]. However, the impact of C5aR/C5a axis activation in ATTRV30M amyloidosis remains to be clarified.

### 4.4. Chemotherapy-Induced Peripheral Neuropathy

CIPN is the most common neurologic complication of chemotherapy, often limiting the efficacy of cancer treatments [81]. Between 30% and 40% of patients receiving chemotherapy are reported to experience CIPN, and this number is expected to grow as more aggressive pharmacological agents emerge and survival rates increase [82]. The incidence of CIPN varies from 10% to 100%, depending upon the specific anticancer drug or drug combination administered and upon the dosing regimen [83]. CIPN causes pain, sensory loss and poor dexterity, with a significant impact on patient quality of life. When pain is too severe, a change in chemotherapy regimen may be required, with risk of reducing the therapeutic efficacy, or patients may choose to discontinue the treatment [84]. For example, both oxaliplatin and paclitaxel, two widely used chemotherapeutics, have been shown to cause neurotoxicity and alterations in sensory neurons, triggering CIPN [85,86].

Current CIPN management is far from satisfactory, and this is largely due to an inadequate understanding of the complexity of CIPN pathophysiology. The main neurobiological mechanisms involved in CIPN include impaired immune cell signalling and ion channel expression, neurotoxicity, mitochondrial dysfunction, and axon degeneration [87]. Emerging evidence suggests that the immune system and immune-mediated neuro-inflammation are crucial events in the development of CIPN [88]. In particular, a recent study reported that in paclitaxel-induced mechanical allodynia the complement cascade is reduced in C3 KO rats compared to WT animals, and that MAC is tightly involved in the damage of neuronal cells, suggesting that complement may be a novel target for the treatment of CIPN [89]. However, since C3 deficiency almost completely abolishes the release of C5a and MAC, which are crucial for the physiological protection against pathogens, and thus exposes the patient to an increased risk of infections and related side effects, the role of C5a-mediated signalling in CIPN models should be further investigated with the aim to develop more targeted treatment.

## 5. C5 Component Targeted Therapies

Several findings indicate that C5 exerts a potent nociceptive activity and contributes to the genesis of both inflammatory and neuropathic pain [8,90,91]. Identifying modulators able to inhibit the C5 cascade may, thus, pave the way toward the development of improved treatments for neuropathic pain.

To date, advances have been made in the characterization of molecular mechanisms underlying C5 signalling, and several C5 inhibitors and modulators have been developed [92]. Currently, the most encouraging therapeutic approaches include monoclonal antibodies modulating C5 component pathways or peptidomimetics that mimic specific portions of C5aR, as well as small molecules targeting specific C5aR binding sites, and potent and selective C5aR inhibitors.

In the following sections, we describe C5- and C5aR-targeting drugs and discuss the importance of the allosteric mechanism of action of novel C5aR antagonists as well as their potential in the treatment of neuropathies such as those previously described.

## 6. Targeting C5

Most of the new molecules identified and developed against C5 are monoclonal antibodies. Among these, eculizumab (Soliris^®^), a monoclonal antibody against C5, binds to C5 and blocks its cleavage into C5a and C5b, ultimately preventing the formation of the MAC matrix [93,94,95] (Table 1).

Eculizumab was the first drug approved by Food and Drug Administration (FDA) [96] for the treatment of paroxysmal nocturnal haemoglobinuria (PNH) [97] and atypical haemolytic uremic syndrome (aHUS) [98], and it is currently under clinical evaluation for its application in several other diseases and conditions, such as GBS [99], neuromyelitis optica [100], kidney and liver transplant rejection [101], systemic lupus erythematosus [102], chemotherapy-induced thrombotic microangiopathy (TMA) [103], and generalized myasthenia gravis [104]. Biosimilars of eculizumab, such as ABP 959 (NCT03818607), BCD-148 (NCT04060264) and SB12 (NCT04058158), are in phase 3 of evaluation for treatment of patients with PNH.

Since treatment with eculizumab was shown to be not completely effective in PNH, new optimized antibodies, follow up compounds of eculizumab, have been developed in recent years [105]. Among these, crovalimab, a novel anti-C5 sequential monoclonal antibody recycling technology, is currently under investigation for PNH (NCT03157635) as promising results were obtained after its administration once every 4 weeks in patients with PNH [106]. Nomacopan, formerly known as rVA576 (Coversin), a second-generation C5 complement inhibitor, is a bi-functional recombinant small protein which is currently in phase 3 of clinical development for PNH (NCT03588026). Like eculizumab, nomacopan prevents cleavage and activation of C5 but it binds C5 at a different site, and this makes it a potentially useful agent to treat PNH patients who are resistant to eculizumab therapy because of C5 genetic variants [107]. Ravulizumab (Ultomiris®), also known as ALXN1210, is a humanized monoclonal antibody designed to bind to and prevent the activation of C5. The molecule is a long-acting C5 inhibitor, which was engineered from eculizumab with increased elimination and half-life, thus allowing an extended dosing interval from two to eight weeks [108]. It is indicated for the treatment of patients with PNH (NCT03406507), aHUS (NCT01522183) and for the inhibition of complement-mediated TMA [108]. Ravulizumab is under current development for amyotrophic lateral sclerosis (ALS) (NCT04248465), neuromyelitis optica spectrum disorder (NMOSD) (NCT04201262) and severe acute respiratory syndrome coronavirus 2 (SARS-CoV-2) infection (COVID-19) (NCT04570397). Zilucoplan is a small (3.5 kDa), 15–amino acid macrocyclic peptide that binds to C5 with high affinity and specificity [109], and it is currently under clinical development for the generalized myasthenia gravis (NCT04115293) and COVID-19 (NCT04590586). Pozelimab is a fully human anti-complement C5, designed for the potential treatment of PNH and CD-55 deficient protein-losing enteropathy (CHAPLE disease) (NCT04209634). The molecule showed to be more potent in decreasing C5 levels and hemolysis in humans and animal models compared to eculizumab and ravulizumab [110], pushing its further investigation for the treatment of PNH and other complement-mediated diseases. Finally, a PEGylated anti-C5 aptamer, avacincaptad pegol sodium (Zimura^®^), is also under active development in a phase 2/3 trial in patients with geographic atrophy secondary to dry age-related macular degeneration (AMD) [111].

## 7. Targeting C5aR

One of the most studied C5aR antagonists, avacopan (Vynpenta^®^), previously called CCX-168, is an orally available selective small molecule optimized for the treatment of orphan and rare renal conditions, primarily anti-neutrophil cytoplasmic auto-antibody (ANCA)-associated vasculitis [112]. Based on promising results from the phase 3 ADVOCATE trial, FDA approval was requested in July 2020.

Avdoralimab, also known as IPH5401, is a human monoclonal antibody that targets C5aR expressed on neutrophils and myeloid-derived suppressor cells (MDSCs), reducing the release of pro-inflammatory factors and the proliferation of cancer cells [113,114,115]. Blocking C5a/C5aR1 axis is now being investigated in clinical trials for the treatment of non-small cell lung cancer (NCT03665129), advanced/metastatic cancer and SARS-CoV-2 infection (NCT04333914), and bullous pemphigoid (NCT04563923). As previously mentioned, no C5aR inhibitors are currently under development for the treatment of neuropathies.

## 8. Allosteric C5a Receptor Inhibitors

Over the last few decades, a plethora of molecules targeting C5, C5a, and/or C5a receptors have been described [95,116]. However, despite the number of preclinical and clinical studies reported, few C5 and C5a receptor inhibitors have been tested in clinical trials and approved for clinical use, mainly as a result of unclear disease mechanisms and unwanted side effects [117].

The dysregulation of complement components contributes to the pathogenesis of various diseases, including neuropathy [21]. Although targeting C5 cleavage was shown to be the most successful strategy in complement-targeted therapies [92,95], developing more selective approaches targeting only one of the two products deriving from C5 cleavage and exploiting alternative mechanisms could be preferable. Considering the crucial role that complement system plays in the innate defense against common pathogens and in immune signalling, caution should be taken when using complement-targeted therapeutics, especially in the treatment of chronic diseases [118]. As observed in patients treated with anti-complement therapy, prolonged complement suppression through the chronic use of immunomodulators can increase susceptibility of patients to certain pyogenic infection or result in adverse events [119]. Eculizumab, for example, impairs host defense against meningococcal infection and increases susceptibility to various infectious pathogens, making vaccination necessary two weeks before the first administration of the compound to avoid this risk [120,121]. Thus, a good and clear safety profile must be a priority in the development of new candidate drugs.

To date, many strategies have been proposed to design more promising complement-specific drugs with the aim of improving the treatment of complement component-mediated disorders. Several studies have suggested a two-site binding mechanism for C5a, with C-terminal segment responsible for activating C5aR2, leading to the development of C5aR antagonists such as peptidomimetics mimicking the structure of C-terminal segment of C5a [122]. The cyclic hexapeptide PMX-53 was developed as a competitive antagonist of C5aR1, with activity at nanomolar concentrations [123]. The molecule blocks C5aR1 at an earlier stage of the immune and inflammatory process compared to the targets of currently available anti-inflammatory drugs and was found efficacious when given by intravenous, intraperitoneal, and subcutaneous injection, as well as by transdermal administration in several rat models of inflammatory diseases [123]. In humans, PMX-53 resulted safe and well tolerated both as an oral formulation for the treatment of inflammatory disorders and as a topical formulation for the treatment of rheumatoid arthritis and psoriasis [124]. Discouragingly, the molecule displayed several drawbacks, mainly due to its peptide nature and low bioavailability, which significantly limit its use. PMX-205, a lipophilic analogue of PMX-53, was found to have greater in vivo efficacy and stability than its parent molecule [123], and has thus been suggested as an ideal drug candidate for several human diseases, including neurological disorders. Many other small molecules with improved drug-like properties exerting reversible and competitive action on C5aR were subsequently developed [122,125]. However, their limited translation to humans is probably due to an insufficient understanding of disease mechanisms and the main parameters influencing their efficacy, such as lifetime of the receptor-ligand complex, which determines the duration of the inhibitory action on C5aR functions [125,126].

An alternative strategy to design more specific C5aR inhibitors could be to develop allosteric modulators of C5aR that, due to their structurally-driven design, generally show improved drug-likeness properties and consequently improved safety characteristics [94,127]. In this context, promising results have been achieved with the non-competitive allosteric inhibitor DF2593A in a SNI animal model of chronic neuropathic pain [40], in the pathogenesis of which C5a/C5aR axis is known to play a role. The molecule was originally identified from a drug discovery approach based on a bio- and chemo-informatic platform targeting GPCRs, including C5aR, aimed at selecting and characterizing new chemical classes of allosteric modulator. Due to the unavailability of the crystal structure of C5aR protein, the design of this novel class of allosteric small-molecular-weight C5aR inhibitors was conducted using a homology modelling approach by exploiting combined information on structural and functional features of allosteric sites in homologous chemokine receptors [40]. Starting from the results on DF2593A, additional medicinal chemistry, in vitro, and in vivo pharmacological studies led to the identification and characterization of novel chemical classes of C5aR allosteric inhibitors, thus allowing the selection of a second generation and new lead compound, DF3966A, with optimized pharmacokinetic and safety profiles but with the same activity and selectivity previously observed [128].

With these data, a new paradigm is emerging for the development of complement-targeted drugs in which the use of allosteric C5aR inhibitors could represent a new strategy to treat and mitigate pain in different neuropathies induced by a dysregulation of complement component response.

## 9. Conclusions

Neuropathy-related pain is a challenging condition to treat and current therapeutic strategies fail to achieve adequate pain relief primarily because the main causes of pain are complex, knowledge of the underlying mechanisms is poor, and the selection of treatment options is often incorrect [29]. Even if patients can find some relief when the underlying cause is addressed, they require careful monitoring and follow-up. Developing drugs to treat any type of nerve pain or to prevent it from getting worse relies on a clear understanding of the mechanisms involved.

The field of complement-targeted therapeutics is developing rapidly and holds promise for the treatment of several neuropathies in the pathogenesis of which the complement is an important factor. Studies investigating a variety of novel drugs—either approved or in late-stage of clinical development—have confirmed that anti-complement approaches can provide new insights into disease mechanisms and enhanced therapies in a growing number of diseases [117,129]. However, several challenges remain to be addressed. For example, the different role of C5aR1 and C5aR2 inhibition in these diseases is poorly understood, and inhibition of C5 activation for prolonged time periods results in increased susceptibility to infectious diseases and other undesired effects. By gaining a greater understanding of the specific mechanisms underlying drug action and optimizing functional selectivity, the use of allosteric inhibitors could speed up the development of anti-complement therapeutic strategies and potentially yield new complement-targeted therapies to treat neuropathic diseases for which no cure is yet available.

## Figures and Tables

**Table 1:** List of compounds under development targeting the complement system.

Drug	Indication	Trial Phase/Study Type	Recruitment Status	NCT Number
Eculizumab, C5-targeting antibody	PNH	Phase 2/3	Only recruiting Trials are listed	NCT04079257
	aHUS-associated multiple organ dysfunction syndrome in hematopoietic stem cell transplant recipients	Phase 2		NCT03518203
	Refractory GMG	Phase 3		NCT03759366
	COVID-19	Phase 2		NCT04346797
	CHAPLE disease	Prospective Cohort		NCT03950804
	PNH	Phase 3		NCT04432584
	PNH	Phase 3		NCT03818607
	PNH	Phase 3		NCT04434092
	End Stage Liver Disease	Phase 1		NCT03468140
	PNH	Observational Study		NCT01374360
	aHUS	Observational Study		NCT01522183
	Neuromyelitis optica spectrum disorder	Phase 2/3		NCT04155424
ABP959, C5-targeting antibody (eculizumab biosimilar)	PNH	Phase 3	Recruiting	NCT03818607
SB12 C5-targeting antibody (eculizumab biosimilar)	PNH	Phase 3	Active, not recruiting	NCT04058158
BCD-148, C5-targeting antibody (eculizumab biosimilar)	PNH	Phase 3	Active, not recruiting	NCT04060264
	Healthy subjects	Phase 1	Completed	NCT04027803
Nomacopan, C5-targeting protein	PNH, aHUS	Phase 3	Recruiting	NCT03829449
	BP	Phase 2	Completed	NCT04035733
	PNH	Phase 3	Completed	NCT03588026
	PNH	Phase 2	Enrolling by invitation	NCT03427060
	PNH	Phase 2	Completed	NCT02591862
	AKC	Phase 1/2	Active, not recruiting	NCT04037891
Ravulizumab, C5-targeting antibody	COVID-19, thrombotic microangiopathies acute kidney injury	Phase 3	Only recruiting Trials are listed	NCT04570397
	Neuromyelitis optica Neuromyelitis optica spectrum disorder	Phase 3		NCT04201262
	TMA	Phase 3		NCT04557735
	ALS	Phase 3		NCT04248465
	COVID-19 severe pneumonia, acute lung injury, acute respiratory distress syndrome pneumonia, Viral	Phase 3		NCT04369469
	TMA	Phase 3		NCT04543591
	PNH	Phase 3		NCT03406507
	COVID-19	Phase 4		NCT04390464
	Atypical hemolytic-uremic syndrome	Observational Study		NCT01522183
	PNH	Observational Study		NCT01374360
	PNH	Phase 3		NCT04432584
Pozelimab, C5-targeting antibody	Healthy volunteer	Phase 1	Active, not recruiting	NCT04491838
	Healthy subjects (in combination with cemdisiran)	Phase 1	Recruiting	NCT04601844
	CHAPLE	Phase 2/3	Recruiting	NCT04209634
Crovalimab, C5-targeting antibody	PNH	Phase 3	Not yet recruiting	NCT04654468
	PNH	Phase 3	Recruiting	NCT04432584
	PNH	Phase 3	Recruiting	NCT04434092
	PNH	Phase 1/2	Active, not recruiting	NCT03157635
Cemdisiran, C5 targeting RNAi therapeutic	IgAN, Berger disease, glomerulonephritis	Phase 2	Recruiting	NCT03841448
	Healthy	Phase 1	Recruiting	NCT04601844
	aHUS	Phase 2	Withdrawn	NCT03303313
	PNH	Phase 1	Completed	NCT02352493
	TMA	Phase2	Not yet recruiting	NCT03999840
IFX-1, C5a targeting antibody	Pyoderma gangrenosum	Phase 2	Recruiting	NCT03971643
	Severe COVID-19 pneumonia	Phase 2/3	Recruiting	NCT04333420
	GPA, MPA	Phase 2	Active, not recruiting	NCT03712345
	GPA, MPA	Phase 2	Active, not recruiting	NCT03895801
	HS	Phase 2	Completed	NCT03487276
	HS	Phase 2	Completed	NCT03001622
	Systemic inflammatory response syndrome, C.surgical procedure, cardiac	Phase 2	Completed	NCT02866825
	Severe sepsis, septic shock	Phase 2	Completed	NCT02246595
	Drug Safety	Phase 1	Completed	NCT01319903
BDB-001, C5a targeting antibody	COVID-19 pneumonia	Phase 2/3	Recruiting	NCT04449588
	Solid tumor	Phase 1	Recruiting	NCT04196530
	Solid tumor	Phase 1	Recruiting	NCT03486301
	Solid tumor, pancreatic cancer, virus associated tumors, non-small cell lung cancer, melanoma, bladdder cancer, triple negative breast cancer	Phase 2	Not yet recruiting	NCT03915678
ALXN1007, C5a targeting antibody	Antiphospholipid (aPL)-positive	Phase 2	Terminated	NCT02128269
	Acute (GVHD), GIGVHD	Phase 2	Terminated	NCT02245412
	Healthy	Phase 1	Completed	NCT01883544
	Healthy	Phase 1	Completed	NCT01454986
Zilucoplan, C5-targeting peptide	GMG	Phase 3	Recruiting	NCT04115293
	ALS	Phase 2/3	Enrolling by invitation	NCT04436497
	ALS	Phase 2/3	Recruiting	NCT04297683
	GMG	Phase 3	Recruiting	NCT04225871
	COVID-19	Phase 2	Active, not recruiting	NCT04382755
	COVID-19	Phase 3	Recruiting	NCT04590586
	IMNM	Phase 2	Recruiting	NCT04025632
	PNH	Phase 2	Active, not recruiting	NCT03225287
	PNH	Phase 2	Completed	NCT03030183
	PNH	Phase 2	Completed	NCT03078582
	GMG	Phase 2	Completed	NCT03315130
Tesidolumab, C5-targeting protein	Wet AMD, Exudative MD	Phase 2	Completed	NCT01535950
	PNH	Phase 2	Active, not recruiting	NCT02534909
	Transplant associate microangiopathy	Phase 2	Terminated	NCT02763644
	Kidney transplantation	Phase 1	Completed	NCT02878616
	Geographic atrophy, AMD	Phase 2	Completed	NCT01527500
	Advanced AMD	Phase 1	Completed	NCT01255462
	Geographic atrophy	Phase 1	Completed	NCT02515942
	Neovascular AMD	Phase 2	Terminated	NCT01624636
	Non-infectious intermediate uveitis, non-infectious posterior uveitis, non-infectious panuveitis	Phase 2	Completed	NCT01526889
Avacopan, C5aR1-targeting small molecule	ANCA-associated vasculitis	Phase 3	Completed	NCT02994927
	ANCA-associated vasculitis	Phase 2	Completed	NCT02222155
	Vasculitis	Phase 2	Completed	NCT01363388
	aHUS	Phase 2	Terminated	NCT02464891
	IgAN	Phase 2	Completed	NCT02384317
	Hidradenitis, suppurativa acne inversa	Phase 2	Active, not recruiting	NCT03852472
	C3 Glomerulopathy	Phase 3	Recruiting	NCT03301467
Avacincaptad, C5-targeting oligonucleotide	Geographic atrophy secondary to AMD	Phase 2/3	Completed	NCT02686658
	IPCV	Phase 2	Completed	NCT02397954
	AMD	Phase 2	Completed	NCT03362190
	IPCV	Phase 2	Withdrawn	NCT03374670
	Stargardt disease 1	Phase 2	Recruiting	NCT03364153
	Geographic atrophysecondary to MD	Phase 3	Recruiting	NCT04435366
Avdoralimab, C5aR1-targeting antibody	BP	Phase 2	Recruiting	NCT04563923
	COVID-19, infection advanced and metastatic hematological or solid tumor	Phase 2	Recruiting	NCT04333914
	COVID-19	Phase 2	Active, not recruiting	NCT04371367
	Advanced solid tumors	Phase 1	Recruiting	NCT03665129

Abbreviations: NCT, national clinical trial; aHUS, atypical hemolytic uremic syndrome; AKC, atopic keratoconjunctivitis; ALS, amyotrophic lateral sclerosis; AMD, age-related macular degeneration; ANCA, anti-neutrophil cytoplasmic autoantibody; BP, Bullous pemphigoid; CHAPLE, complement hyperactivation, angiopathic thrombosis, protein losing enteropathy; COVID-19, coronavirus disease 2019; GBS, Guillain-Barré syndrome; GIGVHD, gastrointestinal graft versus host disease; GMG, generalized myasthenia gravis; GPA, granulomatosis with polyangiitis IgAN, IgA nephropathy; GVHD, graft versus host disease; HS, hidradenitis suppurativa; IMNM, immune-mediated necrotizing myopathy; IPCV, idiopathic polypoidal choroidal vasculopathy; MD, macular degeneration; MPA, microscopic polyangiitis; PNH, paroxysmal nocturnal hemoglobinuria; TMA, complement-mediated thrombotic microangiopathy.

## Data Availability

Not applicable.

## References

[1] Nesargikar P., Spiller B., Chavez R. (2012). The complement system: History, pathways, cascade and inhibitors. Eur. J. Microbiol. Immunol..

[2] Merle N.S., Church S.E., Fremeaux-Bacchi V., Roumenina L.T. (2015). Complement System Part I—Molecular Mechanisms of Activation and Regulation. Front. Immunol..

[3] Ricklin D., Lambris J.D. (2013). Complement in immune and inflammatory disorders: Pathophysiological mechanisms. J. Immunol..

[4] Manthey H.D., Woodruff T.M., Taylor S.M., Monk P.N. (2009). Complement component 5a (C5a). Int. J. Biochem. Cell Biol..

[5] Guo R.F., Ward P.A. (2005). Role of C5a in inflammatory responses. Annu. Rev. Immunol..

[6] Heesterbeek D.A., Bardoel B.W., Parsons E.S., Bennett I., Ruyken M., Doorduijn D.J., Gorham R.D., Berends E.T., Pyne A.L., Hoogenboom B.W. (2019). Bacterial killing by complement requires membrane attack complex formation via surface-bound C5 convertases. EMBO J..

[7] Fritzinger D.C., Benjamin D.E. (2016). The Complement System in Neuropathic and Postoperative Pain. Open Pain J..

[8] Quadros A.U., Cunha T.M. (2016). C5a and pain development: An old molecule, a new target. Pharmacol. Res..

[9] Merle N.S., Noe R., Halbwachs-Mecarelli L., Fremeaux-Bacchi V., Roumenina L.T. (2015). Complement System Part II: Role in Immunity. Front. Immunol..

[10] Mamidi S., Hone S., Kirschfink M. (2017). The complement system in cancer: Ambivalence between tumour destruction and promotion. Immunobiology.

[11] Cedzynski M., Swierzko A.S. (2020). Components of the Lectin Pathway of Complement in Haematologic Malignancies. Cancers.

[12] Mortensen S.A., Sander B., Jensen R.K., Pedersen J.S., Golas M.M., Thiel S., Andersen G.R. (2018). Models of the complement C1 complex. Proc. Natl. Acad. Sci. USA.

[13] Gaboriaud C., Thielens N.M., Gregory L.A., Rossi V., Fontecilla-Camps J.C., Arlaud G.J. (2004). Structure and activation of the C1 complex of complement: Unraveling the puzzle. Trends Immunol..

[14] Zwarthoff S.A., Berends E.T.M., Mol S., Ruyken M., Aerts P.C., Jozsi M., de Haas C.J.C., Rooijakkers S.H.M., Gorham R.D. (2018). Functional Characterization of Alternative and Classical Pathway C3/C5 Convertase Activity and Inhibition Using Purified Models. Front. Immunol..

[15] Kjaer T.R., Jensen L., Hansen A., Dani R., Jensenius J.C., Dobo J., Gal P., Thiel S. (2016). Oligomerization of Mannan-binding Lectin Dictates Binding Properties and Complement Activation. Scand. J. Immunol..

[16] Takahashi M., Iwaki D., Kanno K., Ishida Y., Xiong J., Matsushita M., Endo Y., Miura S., Ishii N., Sugamura K. (2008). Mannose-binding lectin (MBL)-associated serine protease (MASP)-1 contributes to activation of the lectin complement pathway. J. Immunol..

[17] Mortensen S., Kidmose R.T., Petersen S.V., Szilagyi A., Prohaszka Z., Andersen G.R. (2015). Structural Basis for the Function of Complement Component C4 within the Classical and Lectin Pathways of Complement. J. Immunol..

[18] Thurman J.M., Holers V.M. (2006). The central role of the alternative complement pathway in human disease. J. Immunol..

[19] Chen Z.A., Pellarin R., Fischer L., Sali A., Nilges M., Barlow P.N., Rappsilber J. (2016). Structure of Complement C3(H2O) Revealed By Quantitative Cross-Linking/Mass Spectrometry And Modeling. Mol. Cell Proteom..

[20] Hernandez M.X., Namiranian P., Nguyen E., Fonseca M.I., Tenner A.J. (2017). C5a Increases the Injury to Primary Neurons Elicited by Fibrillar Amyloid Beta. ASN Neuro.

[21] Ramaglia V., Daha M.R., Baas F. (2008). The complement system in the peripheral nerve: Friend or foe?. Mol. Immunol..

[22] Dalakas M.C., Alexopoulos H., Spaeth P.J. (2020). Complement in neurological disorders and emerging complement-targeted therapeutics. Nat. Rev. Neurol..

[23] Hughes R.A. (2002). Peripheral neuropathy. BMJ.

[24] Costigan M., Scholz J., Woolf C.J. (2009). Neuropathic pain: A maladaptive response of the nervous system to damage. Annu. Rev. Neurosci..

[25] Reichling D.B., Levine J.D. (2011). Pain and death: Neurodegenerative disease mechanisms in the nociceptor. Ann. Neurol..

[26] Jin H.W., Flatters S.J., Xiao W.H., Mulhern H.L., Bennett G.J. (2008). Prevention of paclitaxel-evoked painful peripheral neuropathy by acetyl-L-carnitine: Effects on axonal mitochondria, sensory nerve fiber terminal arbors, and cutaneous Langerhans cells. Exp. Neurol..

[27] Head K.A. (2006). Peripheral neuropathy: Pathogenic mechanisms and alternative therapies. Altern. Med. Rev..

[28] Hanewinckel R., Ikram M.A., Van Doorn P.A. (2016). Peripheral neuropathies. Handb. Clin. Neurol..

[29] Colloca L., Ludman T., Bouhassira D., Baron R., Dickenson A.H., Yarnitsky D., Freeman R., Truini A., Attal N., Finnerup N.B. (2017). Neuropathic pain. Nat. Rev. Dis. Primers.

[30] Girach A., Julian T.H., Varrassi G., Paladini A., Vadalouka A., Zis P. (2019). Quality of Life in Painful Peripheral Neuropathies: A Systematic Review. Pain Res. Manag..

[31] Liampas A., Rekatsina M., Vadalouca A., Paladini A., Varrassi G., Zis P. (2020). Non-Pharmacological Management of Painful Peripheral Neuropathies: A Systematic Review. Adv. Ther..

[32] Liampas A., Rekatsina M., Vadalouca A., Paladini A., Varrassi G., Zis P. (2020). Pharmacological Management of Painful Peripheral Neuropathies: A Systematic Review. Pain Ther..

[33] Nabizadeh J.A., Manthey H.D., Panagides N., Steyn F.J., Lee J.D., Li X.X., Akhir F.N.M., Chen W., Boyle G.M., Taylor S.M. (2019). C5a receptors C5aR1 and C5aR2 mediate opposing pathologies in a mouse model of melanoma. FASEB J..

[34] Allegretti M., Moriconi A., Beccari A.R., Di Bitondo R., Bizzarri C., Bertini R., Colotta F. (2005). Targeting C5a: Recent advances in drug discovery. Curr. Med. Chem..

[35] Li X.X., Lee J.D., Kemper C., Woodruff T.M. (2019). The Complement Receptor C5aR2: A Powerful Modulator of Innate and Adaptive Immunity. J. Immunol..

[36] Peng Q., Li K., Sacks S.H., Zhou W. (2009). The role of anaphylatoxins C3a and C5a in regulating innate and adaptive immune responses. Inflamm. Allergy Drug Targets.

[37] Zhang T., Garstka M.A., Li K. (2017). The Controversial C5a Receptor C5aR2: Its Role in Health and Disease. J. Immunol. Res..

[38] Croker D.E., Monk P.N., Halai R., Kaeslin G., Schofield Z., Wu M.C., Clark R.J., Blaskovich M.A., Morikis D., Floudas C.A. (2016). Discovery of functionally selective C5aR2 ligands: Novel modulators of C5a signalling. Immunol. Cell Biol..

[39] Wiese A.V., Ender F., Quell K.M., Antoniou K., Vollbrandt T., Konig P., Kohl J., Laumonnier Y. (2017). The C5a/C5aR1 axis controls the development of experimental allergic asthma independent of LysM-expressing pulmonary immune cells. PLoS ONE.

[40] Moriconi A., Cunha T.M., Souza G.R., Lopes A.H., Cunha F.Q., Carneiro V.L., Pinto L.G., Brandolini L., Aramini A., Bizzarri C. (2014). Targeting the minor pocket of C5aR for the rational design of an oral allosteric inhibitor for inflammatory and neuropathic pain relief. Proc. Natl. Acad. Sci. USA.

[41] Shutov L.P., Warwick C.A., Shi X., Gnanasekaran A., Shepherd A.J., Mohapatra D.P., Woodruff T.M., Clark J.D., Usachev Y.M. (2016). The Complement System Component C5a Produces Thermal Hyperalgesia via Macrophage-to-Nociceptor Signaling That Requires NGF and TRPV1. J. Neurosci..

[42] Griffin R.S., Costigan M., Brenner G.J., Ma C.H., Scholz J., Moss A., Allchorne A.J., Stahl G.L., Woolf C.J. (2007). Complement induction in spinal cord microglia results in anaphylatoxin C5a-mediated pain hypersensitivity. J. Neurosci..

[43] Liang D.Y., Li X., Shi X., Sun Y., Sahbaie P., Li W.W., Clark J.D. (2012). The complement component C5a receptor mediates pain and inflammation in a postsurgical pain model. Pain.

[44] Clark J.D., Qiao Y., Li X., Shi X., Angst M.S., Yeomans D.C. (2006). Blockade of the complement C5a receptor reduces incisional allodynia, edema, and cytokine expression. Anesthesiology.

[45] Ting E., Guerrero A.T., Cunha T.M., Verri W.A., Taylor S.M., Woodruff T.M., Cunha F.Q., Ferreira S.H. (2008). Role of complement C5a in mechanical inflammatory hypernociception: Potential use of C5a receptor antagonists to control inflammatory pain. Br. J. Pharmacol..

[46] Hafer-Macko C., Hsieh S.T., Li C.Y., Ho T.W., Sheikh K., Cornblath D.R., McKhann G.M., Asbury A.K., Griffin J.W. (1996). Acute motor axonal neuropathy: An antibody-mediated attack on axolemma. Ann. Neurol..

[47] Kuwabara S., Yuki N. (2013). Axonal Guillain-Barre syndrome: Concepts and controversies. Lancet Neurol..

[48] Jasti A.K., Selmi C., Sarmiento-Monroy J.C., Vega D.A., Anaya J.M., Gershwin M.E. (2016). Guillain-Barre syndrome: Causes, immunopathogenic mechanisms and treatment. Expert Rev. Clin. Immunol..

[49] McGrogan A., Madle G.C., Seaman H.E., de Vries C.S. (2009). The epidemiology of Guillain-Barre syndrome worldwide. A systematic literature review. Neuroepidemiology.

[50] Jacobs B.C., O’Hanlon G.M., Bullens R.W., Veitch J., Plomp J.J., Willison H.J. (2003). Immunoglobulins inhibit pathophysiological effects of anti-GQ1b-positive sera at motor nerve terminals through inhibition of antibody binding. Brain.

[51] Fokke C., van den Berg B., Drenthen J., Walgaard C., van Doorn P.A., Jacobs B.C. (2014). Diagnosis of Guillain-Barre syndrome and validation of Brighton criteria. Brain.

[52] Schaller B., Radziwill A.J., Steck A.J. (2001). Successful treatment of Guillain-Barre syndrome with combined administration of interferon-beta-1a and intravenous immunoglobulin. Eur. Neurol..

[53] Raphael J.C., Chevret S., Hughes R.A., Annane D. (2002). Plasma exchange for Guillain-Barre syndrome. Cochrane Database Syst. Rev..

[54] Meyer zu Horste G., Hartung H.P., Kieseier B.C. (2007). From bench to bedside--experimental rationale for immune-specific therapies in the inflamed peripheral nerve. Nat. Clin. Pract. Neurol..

[55] O’Hanlon G.M., Plomp J.J., Chakrabarti M., Morrison I., Wagner E.R., Goodyear C.S., Yin X., Trapp B.D., Conner J., Molenaar P.C. (2001). Anti-GQ1b ganglioside antibodies mediate complement-dependent destruction of the motor nerve terminal. Brain.

[56] Halstead S.K., O’Hanlon G.M., Humphreys P.D., Morrison D.B., Morgan B.P., Todd A.J., Plomp J.J., Willison H.J. (2004). Anti-disialoside antibodies kill perisynaptic Schwann cells and damage motor nerve terminals via membrane attack complex in a murine model of neuropathy. Brain.

[57] Halstead S.K., Humphreys P.D., Goodfellow J.A., Wagner E.R., Smith R.A., Willison H.J. (2005). Complement inhibition abrogates nerve terminal injury in Miller Fisher syndrome. Ann. Neurol..

[58] Halstead S.K., Zitman F.M., Humphreys P.D., Greenshields K., Verschuuren J.J., Jacobs B.C., Rother R.P., Plomp J.J., Willison H.J. (2008). Eculizumab prevents anti-ganglioside antibody-mediated neuropathy in a murine model. Brain.

[59] McGonigal R., Cunningham M.E., Yao D., Barrie J.A., Sankaranarayanan S., Fewou S.N., Furukawa K., Yednock T.A., Willison H.J. (2016). C1q-targeted inhibition of the classical complement pathway prevents injury in a novel mouse model of acute motor axonal neuropathy. Acta Neuropathol. Commun..

[60] Dalakas M.C. (2011). Medscape. Advances in the diagnosis, pathogenesis and treatment of CIDP. Nat. Rev. Neurol..

[61] Broers M.C., Bunschoten C., Nieboer D., Lingsma H.F., Jacobs B.C. (2019). Incidence and Prevalence of Chronic Inflammatory Demyelinating Polyradiculoneuropathy: A Systematic Review and Meta-Analysis. Neuroepidemiology.

[62] Koller H., Kieseier B.C., Jander S., Hartung H.P. (2005). Chronic inflammatory demyelinating polyneuropathy. N. Engl. J. Med..

[63] Mathey E.K., Park S.B., Hughes R.A., Pollard J.D., Armati P.J., Barnett M.H., Taylor B.V., Dyck P.J., Kiernan M.C., Lin C.S. (2015). Chronic inflammatory demyelinating polyradiculoneuropathy: From pathology to phenotype. J. Neurol. Neurosurg. Psychiatry.

[64] Dalakas M.C., Engel W.K. (1980). Immunoglobulin and complement deposits in nerves of patients with chronic relapsing polyneuropathy. Arch. Neurol..

[65] Hays A.P., Lee S.S., Latov N. (1988). Immune reactive C3d on the surface of myelin sheaths in neuropathy. J. Neuroimmunol..

[66] Quast I., Keller C.W., Hiepe F., Tackenberg B., Lunemann J.D. (2016). Terminal complement activation is increased and associated with disease severity in CIDP. Ann. Clin. Transl. Neurol..

[67] Cakar A., Durmus-Tekce H., Parman Y. (2019). Familial Amyloid Polyneuropathy. Arch. Neurol..

[68] Kato-Motozaki Y., Ono K., Shima K., Morinaga A., Machiya T., Nozaki I., Shibata-Hamaguchi A., Furukawa Y., Yanase D., Ishida C. (2008). Epidemiology of familial amyloid polyneuropathy in Japan: Identification of a novel endemic focus. J. Neurol. Sci..

[69] Ando Y., Nakamura M., Araki S. (2005). Transthyretin-related familial amyloidotic polyneuropathy. Arch. Neurol..

[70] Plante-Bordeneuve V., Said G. (2011). Familial amyloid polyneuropathy. Lancet Neurol..

[71] Rowczenio D., Quarta C.C., Fontana M., Whelan C.J., Martinez-Naharro A., Trojer H., Baginska A., Ferguson S.M., Gilbertson J., Rezk T. (2019). Analysis of the TTR gene in the investigation of amyloidosis: A 25-year single UK center experience. Hum. Mutat..

[72] Manganelli F., Fabrizi G.M., Luigetti M., Mandich P., Mazzeo A., Pareyson D. (2020). Hereditary transthyretin amyloidosis overview. Neurol. Sci..

[73] Ohmori H., Ando Y., Makita Y., Onouchi Y., Nakajima T., Saraiva M.J., Terazaki H., Suhr O., Sobue G., Nakamura M. (2004). Common origin of the Val30Met mutation responsible for the amyloidogenic transthyretin type of familial amyloidotic polyneuropathy. J. Med. Genet..

[74] Ando Y., Coelho T., Berk J.L., Cruz M.W., Ericzon B.G., Ikeda S., Lewis W.D., Obici L., Plante-Bordeneuve V., Rapezzi C. (2013). Guideline of transthyretin-related hereditary amyloidosis for clinicians. Orphanet J. Rare Dis..

[75] Benson M.D. (2013). Liver transplantation and transthyretin amyloidosis. Muscle Nerve.

[76] Hafer-Macko C.E., Dyck P.J., Koski C.L. (2000). Complement activation in acquired and hereditary amyloid neuropathy. J. Peripher. Nerv. Syst..

[77] Fonseca M.I., Zhou J., Botto M., Tenner A.J. (2004). Absence of C1q leads to less neuropathology in transgenic mouse models of Alzheimer’s disease. J. Neurosci..

[78] Pisalyaput K., Tenner A.J. (2008). Complement component C1q inhibits beta-amyloid- and serum amyloid P-induced neurotoxicity via caspase- and calpain-independent mechanisms. J. Neurochem..

[79] Galvan M.D., Foreman D.B., Zeng E., Tan J.C., Bohlson S.S. (2012). Complement component C1q regulates macrophage expression of Mer tyrosine kinase to promote clearance of apoptotic cells. J. Immunol..

[80] Fonseca M.I., Chu S.H., Berci A.M., Benoit M.E., Peters D.G., Kimura Y., Tenner A.J. (2011). Contribution of complement activation pathways to neuropathology differs among mouse models of Alzheimer’s disease. J. Neuroinflamm..

[81] Quasthoff S., Hartung H.P. (2002). Chemotherapy-induced peripheral neuropathy. J. Neurol..

[82] Pike C.T., Birnbaum H.G., Muehlenbein C.E., Pohl G.M., Natale R.B. (2012). Healthcare costs and workloss burden of patients with chemotherapy-associated peripheral neuropathy in breast, ovarian, head and neck, and nonsmall cell lung cancer. Chemother. Res. Pract..

[83] Balayssac D., Ferrier J., Descoeur J., Ling B., Pezet D., Eschalier A., Authier N. (2011). Chemotherapy-induced peripheral neuropathies: From clinical relevance to preclinical evidence. Expert Opin. Drug Saf..

[84] Kolb N.A., Smith A.G., Singleton J.R., Beck S.L., Stoddard G.J., Brown S., Mooney K. (2016). The Association of Chemotherapy-Induced Peripheral Neuropathy Symptoms and the Risk of Falling. JAMA Neurol..

[85] Salat K. (2020). Chemotherapy-induced peripheral neuropathy: Part 1-current state of knowledge and perspectives for pharmacotherapy. Pharmacol. Rep..

[86] Salat K. (2020). Chemotherapy-induced peripheral neuropathy-part 2: Focus on the prevention of oxaliplatin-induced neurotoxicity. Pharmacol. Rep..

[87] Lees J.G., Makker P.G., Tonkin R.S., Abdulla M., Park S.B., Goldstein D., Moalem-Taylor G. (2017). Immune-mediated processes implicated in chemotherapy-induced peripheral neuropathy. Eur. J. Cancer.

[88] Brandolini L., d’Angelo M., Antonosante A., Allegretti M., Cimini A. (2019). Chemokine Signaling in Chemotherapy-Induced Neuropathic Pain. Int. J. Mol. Sci..

[89] Xu J., Zhang L., Xie M., Li Y., Huang P., Saunders T.L., Fox D.A., Rosenquist R., Lin F. (2018). Role of Complement in a Rat Model of Paclitaxel-Induced Peripheral Neuropathy. J. Immunol..

[90] Jang J.H., Clark D.J., Li X., Yorek M.S., Usachev Y.M., Brennan T.J. (2010). Nociceptive sensitization by complement C5a and C3a in mouse. Pain.

[91] Jang J.H., Liang D., Kido K., Sun Y., Clark D.J., Brennan T.J. (2011). Increased local concentration of complement C5a contributes to incisional pain in mice. J. Neuroinflamm..

[92] Ricklin D., Lambris J.D. (2016). New milestones ahead in complement-targeted therapy. Semin. Immunol..

[93] Matis L.A., Rollins S.A. (1995). Complement-specific antibodies: Designing novel anti-inflammatories. Nat. Med..

[94] Schatz-Jakobsen J.A., Zhang Y., Johnson K., Neill A., Sheridan D., Andersen G.R. (2016). Structural Basis for Eculizumab-Mediated Inhibition of the Complement Terminal Pathway. J. Immunol..

[95] Horiuchi T., Tsukamoto H. (2016). Complement-targeted therapy: Development of C5- and C5a-targeted inhibition. Inflamm. Regen..

[96] US Food and Drug Administration (2014). Soliris® (Eculizumab) [Prescribing Information].

[97] Dmytrijuk A., Robie-Suh K., Cohen M.H., Rieves D., Weiss K., Pazdur R. (2008). FDA report: Eculizumab (Soliris) for the treatment of patients with paroxysmal nocturnal hemoglobinuria. Oncologist.

[98] Palma L.M., Langman C.B. (2016). Critical appraisal of eculizumab for atypical hemolytic uremic syndrome. J. Blood Med..

[99] Misawa S., Kuwabara S., Sato Y., Yamaguchi N., Nagashima K., Katayama K., Sekiguchi Y., Iwai Y., Amino H., Suichi T. (2018). Safety and efficacy of eculizumab in Guillain-Barre syndrome: A multicentre, double-blind, randomised phase 2 trial. Lancet Neurol..

[100] Pittock S.J., Berthele A., Fujihara K., Kim H.J., Levy M., Palace J., Nakashima I., Terzi M., Totolyan N., Viswanathan S. (2019). Eculizumab in Aquaporin-4-Positive Neuromyelitis Optica Spectrum Disorder. N. Engl. J. Med..

[101] Tan E.K., Bentall A., Dean P.G., Shaheen M.F., Stegall M.D., Schinstock C.A. (2019). Use of Eculizumab for Active Antibody-mediated Rejection That Occurs Early Post-kidney Transplantation: A Consecutive Series of 15 Cases. Transplantation.

[102] De Holanda M.I., Porto L.C., Wagner T., Christiani L.F., Palma L.M.P. (2017). Use of eculizumab in a systemic lupus erythemathosus patient presenting thrombotic microangiopathy and heterozygous deletion in CFHR1-CFHR3. A case report and systematic review. Clin. Rheumatol..

[103] Schulte-Kemna L., Reister B., Bettac L., Ludwig U., Furst D., Mytilineos J., Bergmann C., van Erp R., Schroppel B. (2020). Eculizumab in chemotherapy-induced thrombotic microangiopathy. Clin. Nephrol. Case Stud..

[104] Dhillon S. (2018). Eculizumab: A Review in Generalized Myasthenia Gravis. Drugs.

[105] Nishimura J.I. (2020). Antibody therapy for paroxysmal nocturnal hemoglobinuria. Rinsho Ketsueki.

[106] Roth A., Nishimura J.I., Nagy Z., Gaal-Weisinger J., Panse J., Yoon S.S., Egyed M., Ichikawa S., Ito Y., Kim J.S. (2020). The complement C5 inhibitor crovalimab in paroxysmal nocturnal hemoglobinuria. Blood.

[107] Schols S., Nunn M.A., Mackie I., Weston-Davies W., Nishimura J.I., Kanakura Y., Blijlevens N., Muus P., Langemeijer S. (2020). Successful treatment of a PNH patient non-responsive to eculizumab with the novel complement C5 inhibitor coversin (nomacopan). Br. J. Haematol..

[108] Rondeau E., Scully M., Ariceta G., Barbour T., Cataland S., Heyne N., Miyakawa Y., Ortiz S., Swenson E., Vallee M. (2020). The long-acting C5 inhibitor, Ravulizumab, is effective and safe in adult patients with atypical hemolytic uremic syndrome naive to complement inhibitor treatment. Kidney Int..

[109] Howard J.F., Nowak R.J., Wolfe G.I., Freimer M.L., Vu T.H., Hinton J.L., Benatar M., Duda P.W., MacDougall J.E., Farzaneh-Far R. (2020). Clinical Effects of the Self-administered Subcutaneous Complement Inhibitor Zilucoplan in Patients With Moderate to Severe Generalized Myasthenia Gravis: Results of a Phase 2 Randomized, Double-Blind, Placebo-Controlled, Multicenter Clinical Trial. JAMA Neurol..

[110] Latuszek A., Liu Y., Olsen O., Foster R., Cao M., Lovric I., Yuan M., Liu N., Chen H., Zhang Q. (2020). Inhibition of complement pathway activation with Pozelimab, a fully human antibody to complement component C5. PLoS ONE.

[111] Jaffe G.J., Westby K., Csaky K.G., Mones J., Pearlman J.A., Patel S.S., Joondeph B.C., Randolph J., Masonson H., Rezaei K.A. (2020). C5 Inhibitor Avacincaptad Pegol for Geographic Atrophy Due to Age-Related Macular Degeneration: A Randomized Pivotal Phase 2/3 Trial. Ophthalmology.

[112] Merkel P.A., Jayne D.R., Wang C., Hillson J., Bekker P. (2020). Evaluation of the Safety and Efficacy of Avacopan, a C5a Receptor Inhibitor, in Patients With Antineutrophil Cytoplasmic Antibody-Associated Vasculitis Treated Concomitantly With Rituximab or Cyclophosphamide/Azathioprine: Protocol for a Randomized, Double-Blind, Active-Controlled, Phase 3 Trial. JMIR Res. Protoc..

[113] World Health Organization (2019). International Nonproprietary Names for Pharmaceutical Substances (INN): Proposed INN: List 121.

[114] Llaudo I., Fribourg M., Medof M.E., Conde P., Ochando J., Heeger P.S. (2019). C5aR1 regulates migration of suppressive myeloid cells required for costimulatory blockade-induced murine allograft survival. Am. J. Transplant..

[115] Ghouse S.M., Vadrevu S.K., Manne S., Reese B., Patel J., Patel B., Silwal A., Lodhi N., Paterson Y., Srivastava S.K. (2020). Therapeutic Targeting of Vasculature in the Premetastatic and Metastatic Niches Reduces Lung Metastasis. J. Immunol..

[116] Woodruff T.M., Nandakumar K.S., Tedesco F. (2011). Inhibiting the C5-C5a receptor axis. Mol. Immunol..

[117] Harris C.L. (2018). Expanding horizons in complement drug discovery: Challenges and emerging strategies. Semin. Immunopathol..

[118] Ricklin D., Lambris J.D. (2007). Complement-targeted therapeutics. Nat. Biotechnol..

[119] Vignesh P., Rawat A., Singh S. (2017). An Update on the Use of Immunomodulators in Primary Immunodeficiencies. Clin. Rev. Allergy Immunol..

[120] McNamara L.A., Topaz N., Wang X., Hariri S., Fox L., MacNeil J.R. (2017). High Risk for Invasive Meningococcal Disease Among Patients Receiving Eculizumab (Soliris) Despite Receipt of Meningococcal Vaccine. MMWR. Morb. Mortal. Wkly. Rep..

[121] Langereis J.D., van den Broek B., Franssen S., Joosten I., Blijlevens N.M.A., de Jonge M.I., Langemeijer S. (2020). Eculizumab impairs Neisseria meningitidis serogroup B killing in whole blood despite 4CMenB vaccination of PNH patients. Blood Adv..

[122] Liu H., Kim H.R., Deepak R., Wang L., Chung K.Y., Fan H., Wei Z., Zhang C. (2018). Orthosteric and allosteric action of the C5a receptor antagonists. Nat. Struct. Mol. Biol..

[123] Kumar V., Lee J.D., Clark R.J., Noakes P.G., Taylor S.M., Woodruff T.M. (2020). Preclinical Pharmacokinetics of Complement C5a Receptor Antagonists PMX53 and PMX205 in Mice. ACS Omega.

[124] Kohl J. (2006). Drug evaluation: The C5a receptor antagonist PMX-53. Curr. Opin. Mol. Ther..

[125] Dumitru A.C., Deepak R., Liu H., Koehler M., Zhang C., Fan H., Alsteens D. (2020). Submolecular probing of the complement C5a receptor-ligand binding reveals a cooperative two-site binding mechanism. Commun. Biol..

[126] Seow V., Lim J., Cotterell A.J., Yau M.K., Xu W., Lohman R.J., Kok W.M., Stoermer M.J., Sweet M.J., Reid R.C. (2016). Receptor residence time trumps drug-likeness and oral bioavailability in determining efficacy of complement C5a antagonists. Sci. Rep..

[127] Fredslund F., Laursen N.S., Roversi P., Jenner L., Oliveira C.L., Pedersen J.S., Nunn M.A., Lea S.M., Discipio R., Sottrup-Jensen L. (2008). Structure of and influence of a tick complement inhibitor on human complement component 5. Nat. Immunol..

[128] Brandolini L., Grannonico M., Bianchini G., Colanardi A., Sebastiani P., Paladini A., Piroli A., Allegretti M., Varrassi G., Di Loreto S. (2019). The Novel C5aR Antagonist DF3016A Protects Neurons Against Ischemic Neuroinflammatory Injury. Neurotox. Res..

[129] Harris C.L., Pouw R.B., Kavanagh D., Sun R., Ricklin D. (2018). Developments in anti-complement therapy; from disease to clinical trial. Mol. Immunol..

